# Occupational exposure to silica dust in France: an ongoing concern

**DOI:** 10.5271/sjweh.4105

**Published:** 2023-10-01

**Authors:** Laurène Delabre, Marie-Tülin Houot, Adrianna Burtin, Corinne Pilorget

**Affiliations:** 1 Santé publique France, The French Public Health Agency, Saint-Maurice, France.

**Keywords:** crystalline silica, exposure prevalence, job-exposure matrix

## Abstract

**Objectives:**

Crystalline silica is found in many construction materials. Although it is one of the oldest known occupational exposures, new exposure contexts have emerged in recent years. In 2021, France classified work involving exposure to respirable crystalline silica (ie, silica dust) generated by a work process as carcinogenic. In order to assess exposure in the French workforce between 1947 and 2020, we developed a silica job-exposure matrix (JEM) for the Matgéné program.

**Method:**

The JEM was linked with occupational data from different population censuses (1982, 1990, 1999, 2007 and 2017). The proportions and numbers of workers exposed to silica dust in France at these various census time points were estimated and described by sex and industry for 2017.

**Results:**

After decreasing between 1982 and 1999, the proportion of workers exposed to silica dust remained stable at 4%, representing 975 000 workers in 2017. Exposed workers were mostly men (93%), and most worked in the construction industry (64%). This was also the industry where the majority of workers were exposed to a level above the French 8-hour time weighted average occupational exposure limit (TWA-OEL).

**Conclusion:**

A large number of workers in France were still exposed (some highly) to silica dust in 2017 so this agent still poses an occupational health concern. The results of this study provide key information about the continued surveillance of the evolution of exposure to silica dust. In a few years, it will be possible to quantify the impact of the 2021 regulation in terms of proportions and number of workers exposed to silica dust.

Silica is a very common chemical compound and a major component of the Earth’s crust; it can take different forms (crystalline, amorphous, silicates, etc.). As crystalline silica, it is found in many materials extracted from mines and quarries which are used in professional environments. These include sand for concrete and glass production, and clay for ceramic production. Crystalline silica dust is one of the oldest known occupational exposures, and many occupational activities are still subject to it (1). The respiratory diseases caused by respirable crystalline silica dust (‘silica dust’ hereafter) are mainly pneumoconioses (silicosis being the best known), chronic obstructive pulmonary diseases, and bronchopulmonary cancers. In 1997, the International Agency for Research on Cancer (IARC) classified crystalline silica dust as carcinogenic for lung cancer in humans. Other forms of silica are neither classified nor regulated. In France, diseases from workplace exposure to silica dust have been classified as occupational diseases since 1945, and their treatment is fully covered for free by the national health insurance system (silicosis, lung cancers, scleroderma). The first French occupational exposure limits (OEL) to silica dust were defined in 1983.

Studies in France and other countries have determined that occupational exposure to silica dust is still a problem today (1–7). In 2017, a global burden of disease study estimated there were 23 700 (19 100–29 000) cases of silicosis worldwide. Although this number is decreasing, silicosis is still the most common type of pneumoconioses (8). In 2019, the French Agency for Food, Environmental and Occupational Health & Safety (ANSES) published an expert report showing the emergence of new occupational exposure contexts for silica dust linked to the use of artificial stone (eg, kitchen fitters) (9).

In December 2017, the European Union classified work involving exposure to silica dust generated by a work process as carcinogenic (10). This classification led to the implementation of compulsory regulation in France on 1 January 2021 (11). One of the consequences of this is the mandatory monitoring of workplace exposure and the elimination or limiting of the most dangerous work processes.

Job-exposure matrices (JEM) are a very useful tool in occupational epidemiology to assess occupational exposure in a large population and exposure to agents, which workers themselves find difficult to identify. To estimate occupational exposure to silica dust among workers in France, we created a JEM specific to silica dust (silica-JEM hereafter) as part of the Matgéné program, which aimed to create various retrospective population-based JEM for different substances. Previous estimations based on the silica-JEM, and on a sample representative of the working population in France (including occupational history calendars), highlighted that 3.1% of all workers (5.6% of men and 0.3% of women) in the country were exposed to silica dust in 2007. These estimated that 15.6% of men had had at least one job in their occupational lifetime where they were potentially exposed to this agent (12).

Linking the silica-JEM – which assesses exposure between 1947 and 2020 – with census data, we investigated the evolution in the proportions and numbers of workers exposed to silica dust in France between 1982 and 2017, and described these proportions according to sex and industry for 2017.

## Method

### Exposure assessment

As part of France’s Matgéné program, two industrial hygienists (IH) developed the silica-JEM to assess exposure to silica dust from 1947 to 2020 for the working population.

In the silica-JEM, jobs (defined as an occupation in an industry) are coded using French and international classifications (see supplementary material, www.sjweh.fi/article/4105). For the present study, only French classifications were used as follows, always at the most precise level: 4-digits PCS1982 and 4-digits PCS2003 for occupations; 4-digits NAF1993, 4-digits NAF2003 and 5-digits NAF2008 for industries (13–17). Nomenclature d’activités française (NAF) is the French adaptation of the European Nomenclature of Economic Activities (NACE) revision 2 and the International Standard Industrial Classification (ISIC) revision 4 (18) (19).

Three exposure indices exist for each job in each JEM version as follows: (i) exposure probability is defined as the proportion of workers exposed to silica dust in their workplace (11 classes); (ii) exposure intensity represents the mean atmospheric concentration of silica dust to which a worker is exposed during the current tasks. It also takes into account the mean atmospheric concentration in the surrounding environment (4 classes). The proportion of exposure due to the tasks and the proportion from the surrounding environment could not be distinguished. Moreover, exposure peaks are included in the intensity assessment. This assessment takes into consideration the collective protective equipment but not the personal protective equipment as it is not possible to know if they are appropriate, properly fitted or even used; (iii) exposure frequency is defined as the percentage of worktime during which the worker is exposed (11 classes); and (iv) the mean exposure level for a specific job during an 8-hour working day can be calculated by multiplying the frequency by the intensity (using the mid-point values of the classes).

The two IH in charge of this project assessed all the combinations (occupation/industry sector/period) considered exposed and the indices. To this end, the IH researched data on work processes (tasks, materials, tools…) to identify all the occupational circumstances with silica exposures and created a database with measurements data found (literature, thesis, open databases…). The measurements helped the expertise by giving information about some tasks or some occupations (mostly on the intensity of the exposure). These data did not give information for every combination and were not used to automatically fill the JEM cells.

For each combination, the IH assessed, by consensus, with the help of the information gathered: the intensity of the exposing tasks, the frequency of exposure and the probability of exposure.

### Study population data

To study the trend of occupational exposure to silica dust in France between 1982 and 2017, we used the National Institute of Statistics and Economic Studies (INSEE) population census data (20), which included information on respondents’ job. These aggregated data describe the numbers of workers in France by occupation, industry, age, sex, and department of residence (a ‘department’ is an administrative level in France similar to a ‘county’) for the years 1982, 1990, 1999, 2007 and 2017. The INSEE census method evolved during the study period. The 1982, 1990 and 1999 censuses were exhaustive and each household was obliged to complete the census form. Since 2004, population censuses have become annual and are based on a rolling 5-year sample (ie, data are renewed only every 5 years). As a result, the 2007 and 2017 censuses take into account data from the annual censuses of 2005 to 2009 and 2015 to 2019, respectively. We did not consider this to be a bias in the present study, as INSEE made adjustments to ensure the data collected from the two different methods could be compared (21). For the oldest census (ie, 1982 and 1990), occupational data codes were cross walked into the NAF1993 classifications using an INSEE table.

### Statistical analysis

Census data were linked with the silica-JEM using the occupation and industry codes and the exposure period corresponding to each census year. The number of persons with a specific job potentially exposed to silica dust was calculated by multiplying the exposure probability provided by the silica-JEM (centre value of the probability class) by the number of workers with the job. The proportion of exposed workers was obtained by dividing the sum of potentially exposed workers by the number of workers in the French working population. A sensitivity interval (SI) was calculated by taking the lower and upper bounds of each probability class. All results regarded workers in metropolitan France (ie, the European territory of France) aged 20–74 years. Exposure indicators were described according to sex and industry group. Results are presented in NACE and ISIC industrial classifications to help comparison with international data. The indicators were also described by occupation, but results are not presented here as there is very little similarity between the French and international classifications for occupation.

The exposure level, representing the 8-hour exposure, was calculated by multiplying the intensity and the frequency, using the mid-point values. This exposure level was considered high when it was >0.1 mg/m^3^ [ie, the French 8-hour time weighted average occupational exposure limit (TWA-OEL)].

## Results

**Figure 1:**
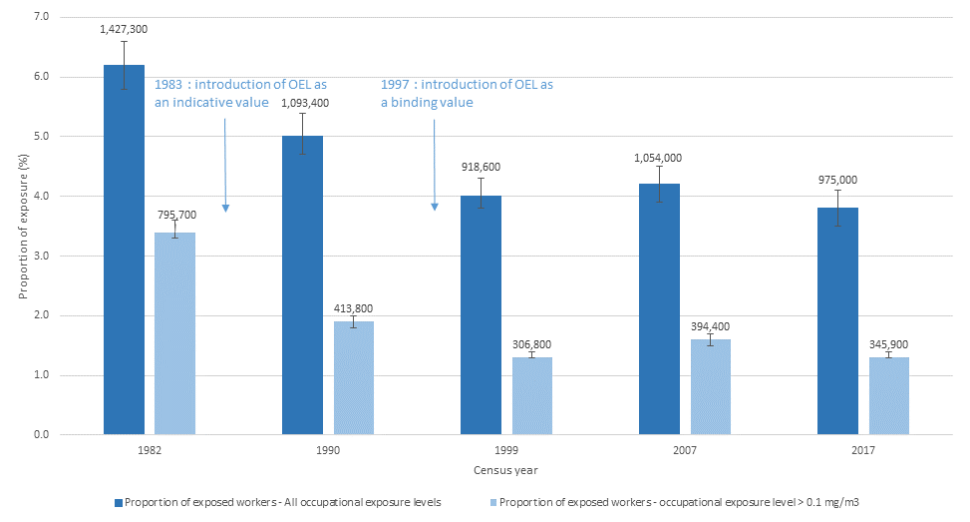
Evolution between 1982 and 2017 of the proportion and the number of workers exposed to silica dust in France.

Using the silica-JEM, we found that the proportion of workers exposed to silica dust aged 20–74 years decreased from 6.2% to 4.0% between 1982 and 1999, and then remained stable (3.8% in 2017) (figure 1). This represents 1 400 000 (SI 1 340 000–1 534 500) exposed workers in 1982 and 975 000 (SI 906 600–1 056 000) in 2017. The proportion of workers with high exposure also decreased over this period, with a 61% reduction (3.4% in 1982 versus 1.3% in 2017).

In 2017, 975 000 workers were exposed to silica dust; of these, 345 900 had a high level of exposure. Most of those exposed were men (93%, 910 000 workers). Among men in 2017, 210 000 workers were exposed in the ‘other specialized construction activities’ sector (ISIC 4390), which includes masonry work (table 1); 97 000 were exposed in the ‘building completion and finishing’ (ISIC 4330) category, while 82 000 and 66 000 were exposed in the ‘electrical installation’ (ISIC 4321) and ‘plumbing, heat and air-conditioning installation’ (ISIC 4322) sectors, respectively. Exposed men were also observed in the ‘general public administration’ (ISIC 8411) and ‘temporary employment agency activities’ (ISIC 7820) sectors. Exposure in these two sectors was also linked to construction tasks. Some smaller industry sectors had fewer exposed men, but the proportion of those exposed in both sectors was high. For example in the ‘manufacture of articles of concrete, cement or plaster’ (ISIC 2395) sector, only 11 000 men were exposed, yet they represented 42% of the workforce in this sector.

**Table 1:** Proportion and number of **male** workers according to activity sector in 2017 as per the ISICS and NACE industrial category classifications. ^a^ [SI=sensitivity interval.]

ISIC 2008	NACE 2008	Total		Number exposed		Proportion exposed		Proportion highly exposed ^b^
		N		N (SI)		% (SI)		% (SI)
4390 - Other specialized construction activities		350 498		210 057 (198 694–224 260)		59.9 (56.7–64.0)		71 (68–75)
4390 - Other specialized construction activities	4391 - Roofing activities	80 042		33 886 (31 250–37 180)		42.3 (39.0–46.5)		8 (8–9)
4390 - Other specialized construction activities	4399 - Other specialised construction activities not classified elsewhere	270 456		176 171 (167 444–187 080)		65.1 (61.9–69.2)		83 (79–87)
4330 - Building completion and finishing		371 785		96 996 (89 635–106 199)		26.1 (24.1–28.6)		38 (37–40)
4330 - Building completion and finishing	4331 - Plastering	44 923		25 766 (24 248–27 664)		57.4 (54.0–61.6)		73 (70–77)
4330 - Building completion and finishing	4332 - Joinery installation	161 075		16 889 (15 498–18 627)		10.5 (9.6–11.6)		32 (31–34)
4330 - Building completion and finishing	4333 - Floor and wall covering	40 369		17 891 (16 507–19 622)		44.3 (40.9–48.6)		8 (7–8)
4330 - Building completion and finishing	4334 - Painting and glazing	110 314		29 988 (27 346–33 289)		27.2 (24.8–30.2)		26 (25–28)
4330 - Building completion and finishing	4339 - Other building completion and finishing	15 103		6463 (6 036–6 997)		42.8 (40.0–46.3)		55 (53–58)
4321 - Electrical installation		184 808		82 409 (77 371–88 706)		44.6 (41.9–48.0)		4 (4–5)
4321 - Electrical installation	4321 - Electrical installation	184 808		82 409 (77 371–88 706)		44.6 (41.9–48.0)		4 (4–5)
4322 - Plumbing, heat and air-conditioning installation		165 395		66 602 (61 434–73 063)		40.3 (37.1–44.2)		8 (7–8)
4322 - Plumbing, heat and air-conditioning installation	4322 - Plumbing, heat and air-conditioning installation	165 395		66 602 (61 434–73 063)		40.3 (37.1–44.2)		8 (7–8)
8411 - General public administration activities		599 193		55 511 (51 138–60 977)		9.3 (8.5–10.2)		6 (6–7)
8411 - General public administration activities	8411 - General public administration activities	599 193		55 511 (51 138–60 977)		9.3 (8.5–10.2)		6 (6–7)
4100 - Construction of buildings		118 683		53 930 (50 857–57 770)		45.4 (42.9–48.7)		63 (60–67)
4100 - Construction of buildings	4110 - Development of building projects	14 526		1176 (1 093–1 279)		8.1 (7.5–8.8)		41 (39–44)
4100 - Construction of buildings	4120 - Construction of residential and non-residential buildings	104 158		52 754 (49 764–56 492)		50.6 (47.8–54.2)		64 (61–67)
4312 - Site preparation		73 067		45 770 (43 500–48 607)		62.6 (59.5–66.5)		38 (37–40)
4312 - Site preparation	4312 - Site preparation	70 574		44 483 (42 286–47 230)		63 (59.9–66.9)		38 (37–41)
4312 - Site preparation	4313 - Test drilling and boring	2493		1287 (1 215–1 377)		51.6 (48.7–55.2)		37 (35–39)
7820 - Temporary employment agency activities		343 120		39 394 (36 814–42 620)		11.5 (10.7–12.4)		57 (54–61)
7820 - Temporary employment agency activities	7820 - Temporary employment agency activities	343 120		39 394 (36 814–42 620)		11.5 (10.7–12.4)		57 (54–61)
4210 - Construction of roads and railways		71 440		33 727 (31 677–36 290)		47.2 (44.3–50.8)		49 (47–52)
4210 - Construction of roads and railways	4211 - Construction of roads and motorways	59 176		29 504 (27 730–31 722)		49.9 (46.9–53.6)		50 (48–53)
4210 - Construction of roads and railways	4212 - Construction of railways and underground railways	6049		1842 (1 720–1 995)		30.5 (28.4–33.0)		41 (39–43)
4210 - Construction of roads and railways	4213 - Construction of bridges and tunnels	6216		2381 (2 227–2 573)		38.3 (35.8–41.4)		38 (36–40)
4220 - Construction of utility projects		46 322		14 364 (13 406–15 560)		31 (28.9–33.6)		37 (35–39)
4220 - Construction of utility projects	4221 - Construction of utility projects for fluids	21 065		9567 (8 959–10 327)		45.4 (42.5–49.0)		40 (38–42)
4220 - Construction of utility projects	4222 - Construction of utility projects for electricity and telecommunications	25 257		4796 (4 447–5 233)		19 (17.6–20.7)		30 (29–32)
2395 - Manufacture of articles of concrete, cement and plaster		26 590		11 302 (10 653–12 112)		42.5 (40.1–45.6)		11 (11–12)
2395 - Manufacture of articles of concrete, cement and plaster	2361 - Manufacture of concrete products for construction purposes	13 558		6562 (6 197–7 017)		48.4 (45.7–51.8)		12 (11–12)
2395 - Manufacture of articles of concrete, cement and plaster	2362 - Manufacture of plaster products for construction purposes	1711		42 (39–45)		2.4 (2.3–2.6)		57 (53–62)
2395 - Manufacture of articles of concrete, cement and plaster	2363 - Manufacture of ready-mixed concrete	8455		3951 (3 723–4 237)		46.7 (44.0–50.1)		9 (9–10)
2395 - Manufacture of articles of concrete, cement and plaster	2364 - Manufacture of mortars	1626		439 (405–481)		27 (24.9–29.6)		2 (2–2)
2395 - Manufacture of articles of concrete, cement and plaster	2365 - Manufacture of fibre cement	260		137 (129–146)		52.6 (49.8–56.2)		3 (3–3)
2395 - Manufacture of articles of concrete, cement and plaster	2369 - Manufacture of other articles of concrete, plaster and cement	981		172 (160–186)		17.5 (16.3–19.0)		62 (59–66)
XXXX - Other industries		10 905 070		199 451 (181 291–217 886)		1.8 (1.7–2.0)		22 (21–24)

^a^ Example of how to read this table: In ISIC 4390, 59.9% of the male workers are exposed to silica (210 057). Among them, 71% are exposed to a level >0.1 mg/m^3^. ^b^ High exposure among those exposed is >0.1 mg/m^3^ (the French 8-hour TWA-OEL).

In terms of high exposure (ie, >0.1 mg/m^3^) in 2017, the sector most concerned was ‘other specialized construction activities’ (ISIC 4390), with 71% (SI 68–75) of exposed male workers having high exposure (148 000 workers).

Despite having a much lower likelihood of exposure to silica dust than men, 65 000 women in France were still exposed in 2017. For the most part, the industrial sectors where exposed women worked were very different from those for exposed men (table 2). What is specific to exposed women is that 29 500 of them worked in a multiplicity of industrial sectors; each of these sectors represented <5% of the total number of exposed women. The ‘general public administration’ sector had 14 000 exposed women, representing a very small proportion of the workforce in this sector (1.4%). The ‘building completion and finishing’ and ‘other specialized construction activities’ sectors (which were the two principal sectors for men) had 3400 exposed female workers in each (7.4% and 10% of the workers of these sectors, respectively). Women were more exposed than men in certain industry sectors. This was the case for example in the ‘manufacture of other porcelain and ceramic products’ sector (2300 exposed female workers, accounting for 54% of the total number of female workers in this sector), and the ‘manufacture of glass’ sector (2100 exposed female workers, accounting for 25% of the total number of female workers in this sector). Just as was observed in men, the ‘other specialized construction activities’ sector had the largest proportion and number of female workers with high exposure (62%, 2073 women).

**Table 2:** Proportion and number of exposed **female** workers according to activity sector in 2017 as per the ISICS and NACE industrial category classifications. ^a^ [SI=senstivity interval.]

ISIC 2008	NACE 2008	Total		Exposed		Proportion exposed		Proportion highly exposed ^b^
		N		N (SI)		% (SI)		% (SI)
8411 - General public administration activities		867 452		14 520 (13 442–15 867)		1.7 (1.5–1.8)		1 (0–1)
8411 - General public administration activities	8411 - General public administration activities	867 452		14 520 (13 442–15 867)		1.7 (1.5–1.8)		1 (0–1)
4330 - Building completion and finishing		47 097		3484 (3 169–3 877)		7.4 (6.7–8.2)		20 (19–21)
4330 - Building completion and finishing	4331 - Plastering	3592		599 (562–645)		16.7 (15.6–18.0)		68 (65–72)
4330 - Building completion and finishing	4332 - Joinery installation	21 865		549 (499–613)		2.5 (2.3–2.8)		17 (17–18)
4330 - Building completion and finishing	4333 - Floor and wall covering	4632		467 (423–522)		10.1 (9.1–11.3)		6 (6–7)
4330 - Building completion and finishing	4334 - Painting and glazing	14 989		1715 (1545–1 927)		11.4 (10.3–12.9)		8 (7–8)
4330 - Building completion and finishing	4339 - Other building completion and finishing	2019		153 (140–170)		7.6 (6.9–8.4)		23 (22–25)
4390 - Other specialized construction activities		33 805		3369 (3159–3631)		10.0 (9.3–10.7)		62 (59-65)
4390 - Other specialized construction activities	4391 - Roofing activities	7571		610 (561–672)		8.1 (7.4–8.9)		7 (6–7)
4390 - Other specialized construction activities	4399 - Other specialised construction activities not classified elsewhere.	26 234		2759 (2598–2959)		10.5 (9.9–11.3)		74 (71–78)
8510 - Pre-primary and primary education		463 960		2339 (2187–2529)		0.5 (0.5–0.5)		0 (0–0)
8510 - Pre-primary and primary education	8510 - Pre-primary education	113 193		684 (639–740)		0.6 (0.6–0.7)		0 (0–0)
8510 - Pre-primary and primary education	8520 - Primary education	350 767		1655 (1548–1789)		0.5 (0.4–0.5)		0 (0–0)
2393 - Manufacture of other porcelain and ceramic products		4242		2321 (2210–2459)		54.7 (52.1–58.0)		0 (0–0)
2393 - Manufacture of other porcelain and ceramic products	2341 - Manufacture of ceramic household and ornamental articles	3313		2091 (1993–2213)		63.1 (60.2–66.8)		0 (0–0)
2393 - Manufacture of other porcelain and ceramic products	2342 - Manufacture of ceramic sanitary fixtures	307		67 (63–72)		21.9 (20.5–23.5)		0 (0–0)
2393 - Manufacture of other porcelain and ceramic products	2343 - Manufacture of ceramic insulators and insulating fittings	127		24 (22–25)		18.6 (17.4–20.0)		20 (19–21)
2393 - Manufacture of other porcelain and ceramic products	2344 - Manufacture of other technical ceramic products	306		38 (36–41)		12.5 (11.8–13.4)		0 (0–0)
2393 - Manufacture of other porcelain and ceramic products	2349 - Manufacture of other ceramic products	189		101 (95–107)		53.2 (50.4–56.8)		0 (0–0)
8610 - Hospital activities		938 140		2185 (2021–2390)		0.2 (0.2–0.3)		1 (1–1)
8610 - Hospital activities	8610 - Hospital activities	938 140		2185 (2021–2390)		0.2 (0.2–0.3)		1 (1–1)
2310 - Manufacture of glass and glass products		8623		2135 (2030–2266)		24.8 (23.5–26.3)		0 (0–0)
2310 - Manufacture of glass and glass products	2311 - Manufacture of flat glass	292		36 (33–40)		12.3 (11.4–13.6)		0 (0–0)
2310 - Manufacture of glass and glass products	2312 - Shaping and processing of flat glass	2664		12 (11–14)		0.5 (0.4–0.5)		0 (0–0)
2310 - Manufacture of glass and glass products	2313 - Manufacture of hollow glass	4080		1598 (1521–1694)		39.2 (37.3–41.5)		0 (0–0)
2310 - Manufacture of glass and glass products	2314 - Manufacture of glass fibres	308		56 (52–60)		18.1 (17.0–19.5)		0 (0–0)
2310 - Manufacture of glass and glass products	2319 - Manufacture and processing of other glass, including technical glassware	1279		433 (412–458)		33.8 (32.2–35.8)		0 (0–0)
8521 - General secondary education		425 791		2058 (1924–2226)		0.5 (0.5–0.5)		0 (0–0)
8521 - General secondary education	8531 - General secondary education	425 791		2058 (1924–2226)		0.5 (0.5–0.5)		0 (0–0)
3250 - Manufacture of medical and dental instruments and supplies		22 518		1994 (1799–2238)		8.9 (8.0–9.9)		4 (4–4)
3250 - Manufacture of medical and dental instruments and supplies	3250 - Manufacture of medical and dental instruments and supplies	22 518		1994 (1799–2238)		8.9 (8.0–9.9)		4 (4–4)
4100 - Construction of buildings		30 447		1533 (1404–1694)		5.0 (4.6–5.6)		42 (40–44)
4100 - Construction of buildings	4110 - Development of building projects	12 520		135 (123–150)		1.1 (1.0–1.2)		13 (13–14)
4100 - Construction of buildings	4120 - Construction of residential and non-residential buildings	17 927		1398 (1281–1544)		7.8 (7.1–8.6)		45 (43–47)
XXXX - Other industries		9 647 299		29 546 (26 759–32 820)		0.3 (0.3–0.3)		8 (8–9)

^a^ Example of how to read this table: In ISIC 8411, 1.7% of female workers are exposed to silica (14 520). Among them, 1% are exposed to a level >0.1 mg/m^3^. ^b^ High exposure among those exposed is >0.1 mg/m^3^ (the French 8-hour TWA-OEL).

Table 3 presents the distribution of exposed workers (men and women together) in 2017 by exposure level. Around 14% of exposed workers had a level of exposure <0.01 mg/m^3^ (a tenth of the TWA OEL) and 36% have a level of exposure >0.1 mg/m^3^.

**Table 3:** Distribution of the exposed workers by exposure level (2017).

Exposure level (mg/m^3^) ^a^	Exposed workers*		Distribution of exposed workers
	N		%
0–0.01	138 950		14
0.01–0.02	176 070		18
0.02–0.05	227 990		23
0.05–0.1	86 130		9
0,1–0.2	297 630		31
0.2–0.3	48 230		5
≥ 0.3	0		0
Total	975 000		100

^a^ Calculated using mid-point values for the intensity and the frequency of exposure

## Discussion

After a decreasing trend until the beginning of the 2000s, the proportion of workers exposed to silica dust in France remained stable at 4% from 2007 to 2017, representing 975 000 people in 2017. The decrease observed between 1982 and 1999 was the consequence of two factors. Firstly, with the establishment of the 1983 and 1997 French OEL regulations, work situations and technical operations changed and, therefore, the intensity, frequency and probability of exposure assessed in the JEM decreased over the period. Secondly, exposure declined as a result of structural change over the studied years in French industry, particularly mines and many industrial activities ceased operations, and as a result there was a large decrease in jobs at risk of exposure. After 2000, the proportion of exposed workers remained stable because there were fewer regulatory and technical changes, and the employment structure of the French workforce remained stable, especially within the construction industry.

Our results for France can be compared to those for other countries. A Finnish study using a similar method and the FINJEM matrix for exposure assessment found that the proportion of workers exposed to silica dust remained stable at 2.2% from 1990 to 2020 [versus 3.8% (3.5–4.1) in our population in 2017] (22). A Swedish study using JEM for exposure assessment also found that the proportion of exposed workers remained stable in Sweden since 2000 (around 3% of exposed workers) (23). A European study published in 2017 estimated that 3–5 million European workers were exposed to silica dust, and the construction industry was the sector with the highest proportion of exposed workers (18.9%) (24). We found similar results, with 38% of workers in this sector exposed (data not shown).

Using both expert knowledge and measurements to assess exposure, a UK study estimated that 590 000 workers were exposed to silica dust between 1990 and 1993 (25), representing 2.3% of the active working population in 1991 (26) (versus 1 000 000 in our study, representing 5% of the French working population). The occupations and industrial sectors mostly concerned by exposure in that study reflect our findings: construction, glass production, ceramic production, and metalworking. Another European study showed that the exposure level decreased from 0.013 to 0.010 mg/m^3^ in Europe between 2000 and 2015, except during the economic crisis of 2008 (27). In our study, the proportion of workers with high exposure also decreased over our study period (1982–2017). An OSHA study estimated that 73 300 American workers were exposed to a level >0.1 mg/m^3^ (twice the recommended OEL in USA) in 2014; this represented <0.1% of the working population in the USA in 2014 (28). In our study, the relative proportion was 1.2%, with exposure primarily in the construction industry. In Canada, an estimated 429 000 workers were exposed in 2016; of these, the vast majority were male workers (94%), which reflects our finding (93%) (29). In Australia, the proportion of exposed workers in 2011 was 6.6%; over half (3.7%) of these had high exposure (ie, >0.1 mg/m^3^) (30).

### Exposure assessment

Some occupational exposures could not be assessed with the silica-JEM as certain job classifications did not always allow specific jobs to be identified, and some jobs had a very low exposure probability or frequency. This was particularly the case for silica dust exposure in agriculture occupations and sectors where the level and probability/frequency of exposure depends on (i) the proportion of silica in the soil and (ii) the type of crop grown (as soil sand content differs for different crops). Available data and the three indices we used (ie, probability, frequency, and intensity) did not allow us to assess a mean level of exposure for agricultural workers, despite exposure being possible during the soil preparation for some crops. Another example is the work of kitchen fitters who cut artificial stone; although the ANSES report (9) indicated that this occupation is one of several new contexts for occupational exposure to silica dust, it could not be identified in the classification we used. It must also be underlined that the production and cutting processes of artificial stone is not an industrial sector in France.

The calculation of the exposure level allows to have a better view of the 8-hour exposure. Some jobs can have a high intensity of exposure due to a task with high exposure, but they might do the task rarely. On the contrary, some can have task with low intensity of exposure, but do it frequently. In this case, their exposure level will be similar.

As presented previously, it was not possible to distinguish exposure from the task versus exposure from the surrounding environment. However, the contribution of the surrounding environment can be important in some industries such as the construction industry (31).

The accuracy of the silica-JEM depends on the occupation and industrial sector classifications used to build it, and because of this it has certain limitations related to the coding of jobs. More specifically, classification codes sometimes group occupations or industries that are heterogeneous in nature and have potentially different exposure risks; these differences make it necessary to define an average exposure for a single code.

In the present study, we used the center values for the classes for the three exposure indices (probability, frequency, and intensity) provided by the silica-JEM to estimate the exposure proportion. Because of this, the results presented in this article are subject to uncertainty; however, this uncertainty is partially mitigated by the SI, which we calculated by taking the lower and upper bounds of each probability class.

Despite their limitations, JEM are still the most suitable tools for assessing exposure in large populations where individual exposure assessment is not possible. The various Matgéné JEM give an historical assessment of exposure to different substances for all occupations and industrial sectors in France.

The indicators we estimated using the silica-JEM provide a detailed description of occupational exposure to silica dust in France in 2017 and the trend in this exposure over the previous 40 years (ie, 1982–2017). In a few years, it will be possible to see whether the 2021 French regulation on silica dust, which classified work involving exposure to silica dust generated by a work process as carcinogenic, has an impact on the proportion of exposed workers. To do this, the silica-JEM will have to be updated.

The indicators we estimated for five time points over several decades using the silica-JEM allowed us to calculate the approximate attributable risk fraction (ARF) to occupational exposure for diseases caused by silica dust. In 2017, 1.1–3% (322–912 cases) of lung cancer cases among men in France and 0–0.1% (6–18 cases) among women, were attributable to occupational exposure to silica dust (32). The total burden of disease is underestimated as the study focused only on cancers and did not include non-malignant respiratory diseases.

Although primarily linked to coal mining and silicosis in the past, silica dust still constitutes an occupational health concern today for many workers in various professional sectors. While work involving exposure to silica dust generated by a work process is now classified as carcinogenic, substituting this substance is difficult, especially for some industry processes such as concrete production. Moreover, it is already present in construction materials used in buildings built to date. Accordingly, France’s 2021 classification, labelling and packaging regulation concerns more the monitoring of exposure than the (essentially impossible) substitution of existing materials. However, according to the French regulation on carcinogenic agents, if the substitution is impossible, other protections must be set up such as limitation of the exposure (working with a wet process), or use of materials with lower silica rate, etc. The Matgéné JEM program actively takes part in the surveillance of the evolution of exposure to silica dust in France by estimating the number of workers currently and formerly exposed in previous decades as well as the industries and occupations concerned.

### Ethic approval

Research approval was not necessary because the aggregated INSEE data used were non-identifiable socioeconomic variables.

## Supplementary material

Supplementary material
