# Supplementary material for: Occupational exposure to silica dust in France: an ongoing concern
Source: Scand J Work Environ Health. 2023 Sep 29;49(7):526–34. doi: 10.5271/sjweh.4105 (PMC10842119; doi:10.5271/sjweh.4105)
Supplement: Supplementary material [file SJWEH-49-526-S001.pdf]

Santé publique France

Occupational exposure to silica dust in France: an ongoing concern[1]

by Laurène Delabre, BSc,[2] Marie-Tülin Houot, MSc, Adrianna Burtin, MSc, Corinne Pilorget, PhD

1. *Free crystalline silica dust job-exposure matrix notebook*
2. *Correspondence to: Laurène Delabre, Santé publique France, The French Public Health Agency, 12 rue du val d'Osne, 94415 Saint-Maurice, France. [E-mail: laurene.delabre@santepubliquefrance.fr]*

JEM update: 17/02/2022

Notebook update: 06/06/2023

1. Objective of this notebook 2
2. Description of the agent 2
a) Silica 2
b) Crystalline silica in occupational environment 2
3. Classification and regulation 3
4. JEM development 4
a) The bibliographic research 4
b) The exposure database 4
5. Classifications used in the JEM 5
6. Exposure assessment indices 5
7. Periods of exposure 7
8. Characteristics of the JEM 8
9. Extract of the JEM 8
10. Publications 9

1. Objective of this notebook

This notebook presents the free crystalline silica dust job-exposure matrix (called silica-JEM) created in the frame of the Matgéné programme. This JEM gives an assessment to respirable free crystalline silica dust for each job considered as exposed, from 1947 to 2020.

It is an update of the existing silica-JEM assessing exposure up to 2007. This update consisted in assessing the exposure for the 2008-2020 period and transcode the silica-JEM in the newest French job classifications for all the periods.

2. Description of the agent

a) Silica

The chemical element Silicium, Si, is a major component of the earth crust (around 25%). It is mainly found as:

• free silica: white or colourless hard solid, with a tetrahedron (SiO_4)

• silicates: SiO_4 tetrahedron as basic pattern too but combined with different metallic oxides (iron, magnesium…).

Silica and silicates can be crystalline (the pattern is regularly repeated in the space) or amorphous (without fixed structure). All these forms can be natural or man-made/synthetic.

Table 1: Examples of different silica forms

Silica forms	Examples
Natural free crystalline silica	quartz, cristobalite, trydimite
Synthetic free crystalline silica	quartz monocrystals
Natural amorphous free silica	diatomaceous earth
Synthetic amorphous free silica	precipitated silica
Natural crystalline silicates	clay, kaolin
Natural amorphous silicates	opal

b) Crystalline silica in occupational environment

The main industries exposed to crystalline silica dust are:

- Mines & quarries
- Construction, cement plant, production of concrete elements
- Production of glass, porcelain pieces, ceramic objects, abrasive objects, false teeth, jewellery…
- Demolition, repair and fabrication of oven in refractory bricks
- Metalworking industries

The rate of silica in materials found in the occupational environment can be very different from a material to another (cf table 2).

Table 2: rate of silica in the different materials

Rate of free crystalline silica	Example of materials
1-10%	Coal and gangue<br>Cement
11-50%	Slate, clay/kaolin<br>Concrete/mortar<br>Metal ores and gangue
≥ 51%	Sand, granite, sandstone…

3. Classification and regulation

- International Agency for Research on Cancer (IARC): crystalline silica was classified as carcinogenic for human (group 1) in 1997, for lung cancer.
- European Union: in December 2017, the European Union classified “work involving exposure to respirable crystalline silica dust generated by a work process” as carcinogenic. This classification was translated in the French law by the order of 26/10/2020. Since January 1st, 2021, these works are considered as carcinogenic in France; this classification will make it compulsory to monitor exposure and to avoid dangerous work process for example this substance must be removed or substituted or, when it is not possible, the exposure must be the lowest as possible.

Occupational exposure limit (OEL)

The order n°2008-244 of the 07/03/2008 is the current regulation in place. The classification as carcinogenic did not change these values.

There are the same values for the 8h TWA-OEL, defined by the order 97-331 of the 10/04/1997 and the one of 21/03/1983.

- 8h TWA-OEL for quartz: 0.1 mg/m^3
- 8h TWA-OEL for trydimite et cristobalite: 0.05 mg/m^3

Occupational disease tables

In France, employees are covered under the general insurance scheme. This system is administered by the occupational accidents and diseases branch of the French social security system and funded by mandatory contributions from employers for each of their establishment.

To be considered occupational in nature and lead to compensation, a disease must appear on one of the occupational disease tables (French database) and be identified as directly linked to the

professional activity by the complementary recognition system for occupational diseases. (source: www.inrs.fr)

Diseases linked to the crystalline silica exposure are listed in table 3.

Table 3: Occupational disease tables linked with crystalline silica exposure

Table number	Name of the table
Number 25 (Employees Health Insurance other than agricultural and self-employed workers)	Diseases linked to mineral dust inhalation containing crystalline silica (quartz, trydimite and cristobalite), crystalline silicates (kaolin, talc), graphite or coal.
Number 22 (Agricultural Health Insurance)	Diseases linked to mineral dust inhalation containing crystalline silica or crystalline silicates

4. JEM development

Nowadays, crystalline silica is the only form to be classified as dangerous for health. Consequently, this JEM assesses the exposure to the free crystalline silica dust only.

The silica-JEM assesses the occupational exposure to respirable free silica dust (diameter >5µm).

The JEM was developed using the expertise of two industrial hygienists (IH) specialised in JEM development and based on bibliographic and metrological researches on the work situation, regulation, exposure data, etc. Both IH validated the assessments.

a) The bibliographic research

A scientific, medical and technical bibliographic research was undertaken and gave information to identify and assess the work situations: regulation, evolution of the exposure level, work technics, etc.

b) The exposure database

An exposure database was also created; it contains 634 measurement results with 22 variables, covering all the study period. Moreover, the data from the Colchic and Scola database were added to these data. Colchic and Sola are two databases handled the INRS, the reference body for the occupational risk prevention in France, containing occupational exposure measurements. (https://www.inrs.fr/media.html?refINRS=BD%201). The measurements helped the expertise by giving information about some tasks or some occupations (mostly on the intensity of the exposure). These data did not give information for every combinations and were not used to automatically fill the JEM cells.

5. Classifications used in the JEM

Assessment to occupational silica exposure were carried out for each couple occupation/industry defined with French and international classification. For industry, we used the French classification NAF ("*Nomenclature des activités française*", 1993, 2003 and 2008) and ISIC (International Standard Industrial Classification from 1975), and for occupation, the French classification PCS ("*Professions et catégories socioprofessionnelles*", 1994 and 2003) and ISCO (International Standard Classification of Occupations from 1968). The most precise level was always used.

Here are the available silica-JEM versions:

- 5-digits ISCO1968 * 4-digits NAF1993;
- 5-digits ISCO1968 * 4-digits ISIC1975;
- 4-digits PCS1994;* 4-digits NAF1993;
- 4-digits PCS2003;* 4-digits NAF2003;
- 4-digits PCS2003;* 5-digits NAF2008.

6. Exposure assessment indices

The JEM assesses exposure between 1947 to 2020, through three indices:

- The exposure probability defined as the proportion of workers exposed in the considered job. It is expressed in percentage, following the classes defined in table 4.

Table 4: probability classes defined in the silica-JEM

Probability classes	Values in the JEM
[1-5]	5
]5-15]	10
]15-25]	20
]25-35]	30
]35-45]	40
]45-55]	50
]55-65]	60
]65-75]	70
]75-85]	80
]85-95]	90
]95-100]	95

- The exposure frequency defined as the percentage of worktime during which the exposing situations occur in the workplace. It is expressed in percentage, following the classes defined in table 5.

Table 5: Frequency classes of the silica-JEM

Frequency classes	Values in the JEM
[1-5]	5
]5-15]	10
]15-25]	20
]25-35]	30
]35-45]	40
]45-55]	50
]55-65]	60
]65-75]	70
]75-85]	80
]85-95]	90
]95-100]	95

- The exposure intensity represents the atmospheric concentration to which the worker is exposed during exposing situations. Exposure intensity represents the mean atmospheric concentration of silica dust to which a worker is exposed during the current tasks. It also takes into account the mean atmospheric concentration in the surrounding environment. The proportion of exposure due to the tasks and the proportion from the surrounding environment could not be distinguished. Moreover, exposure peaks are included in the intensity assessment. This assessment takes into consideration the collective protective equipment but not the personal protective equipment as it is not possible to know if they are appropriate, properly fitted or even used. It is expressed in 4 classes (table 6). Below 0.02mg/m^3, the tasks are considered with no occupational exposure.

Table 6: Intensity classes in the silica-JEM

Values	Values in the JEM	Mid-point values used for the exposure level calculation
[0,02 ; 0,1 mg/m^3 [	1	0,06
[0,1 ;0,5 mg/m^3 [	2	0,3
[0,5 ; 1 mg/m^3 [	3	0,75
≥ 1 mg/m^3	4	1,5

An exposure level can be calculated by multiplying intensity with frequency (using each mid-point values of the classes). It represents the level of exposure on an 8h-worktime. This level can be lower than 0.02 mg/m^3, as it is considered on the entire worktime.

Table 7 presents the exposure level classes.

Table 7: exposure level classes

Exposure level* (mg/m^3)
]0-0,01[
[0,01-0,02[
[0,02-0,05[
[0,05-0,1[
[0,1-0,2[
[0,2-0,3[
≥ 0,3
Total

**Calculated using mid-point values for the intensity and the frequency of exposure*

7. Periods of exposure

The JEM assessment takes into account different parameters, which include the evolution of exposure during time. Different exposure periods have been defined by industry following some criteria (regulation, technical evolution…) (table 7).

Table 7: exposure periods defined in the silica-JEM

Industry	Periods of exposure
Mines	- 1947 to 1960<br>- 1961 to 1980<br>- 1981 to 2020
Quarries	- 1947 to 1960<br>- 1961 to 1995<br>- 1996 to 2020
Other industries and construction industry	- 1947 to 1970<br>- 1971 to 1985<br>- 1986 to 1998<br>- 1999 to 2020

- Mines and quarries:
  - The first period is the same for both because 1960 corresponds to the year of application of the 11/30/1956 instruction which says that good prevention technics needs to be apply
  - For mines, 1980 corresponds to the application of the 12/15/1975 circular about the pneumoconiosis medical prevention
  - For quarries, the 09/02/1994 regulation, defining a referent dust situation led to a decrease in exposure. This regulation didn't have impact on mines because at this time, there were already closed or about to close

- Other industries:
    - 1970 is a period of global improvement in work situation in France in industries
    - 1985 and 1988 are related to the OEL application

The classification as carcinogenic from January 1st, 2021 have no impact on the silica-JEM as the study period ends in 2020.

8. Characteristics of the JEM

In occupational environment, free crystalline silica is mainly quartz and rarely cristobalite. It is very difficult to distinguish these two forms so they were gathered under the term “free crystalline silica” for the assessment.

Several uses of the silica could not be assessed in the JEM because of the classifications used that made exposure probability too low; it is the case for example in the production of glass wool in which silica is used as a basic material.

Some industries are not in the JEM because there were not enough information available to assess exposure. For example, in agriculture, the silica rate in the soil is different from an area to another in France and the task frequency and intensity depend on crops, the farm size etc…The indices used for assessment are not able to take into account these situations and so the jobs in agriculture is not assessed in the JEM.

Some recent studies showed new exposures for the kitchen fitter working on artificial stone. This use cannot be selected because of the job classifications used.

9. Extract of the JEM

Table 8 is an extract of the silica-JEM, in the PCS2003*NAF2008 version.

These lines give the exposure assessment for the unskilled industrial workers (674d) working in the manufacture of concrete products between 1947 and 2020. This job has four different periods with homogenous exposure for each one. During the whole period, these workers had the same probability of exposure: 90 % (between 85 and 95%). The mean intensity of exposure decreased from class 3 (between [0,5 ; 1 mg/m^3 [) to class 1 ([0,02 ; 0,1 mg/m^3 [) in the latest period. The mean frequency of exposure also decreased from 90% (between 85 and 95%) of the working time during 1947-1970 to 50% (between 45 and 55%) during 1999-2020.

Table 8: extract of the silica-JEM.

Occupation code (from the French occupation classification, PCS 2003)	Description of the occupation code	Industry code (from the French industry classification, NAF 2008)	Description of the industry code	Probability of exposure	Intensity of exposure	Frequency of exposure	Starts	Ends
674d	Unskilled industrial workers in metal working, glass and ceramic production, and manufacture of construction material	2361Z	Manufacture of concrete products for construction purposes	90	3	90	1947	1970
674d	Unskilled industrial workers in metal working, glass and ceramic production, and manufacture of construction material	2361Z	Manufacture of concrete products for construction purposes	90	2	70	1971	1985
674d	Unskilled industrial workers in metal working, glass and ceramic production, and manufacture of construction material	2361Z	Manufacture of concrete products for construction purposes	90	1	70	1986	1998
674d	Unskilled industrial workers in metal working, glass and ceramic production, and manufacture of construction material	2361Z	Manufacture of concrete products for construction purposes	90	1	50	1999	2020

10. Publications

The JEM is freely consultable on the website: www.exppro.fr.

The geographic indicators (proportion and numbers of exposed workers), estimated by linking the JEM with census data are consultable on the website: https://geodes.santepubliquefrance.fr/#c=home.

This JEM and its uses, have been published in several articles:

- Delabre L, Houot M, Burtin A et Pilorget C. L'exposition professionnelle à la silice cristalline en France en 2017 : une question toujours d'actualité. Bull Epidémiol Hebd. 2023 ;(1) :16-24. http://beh.santepubliquefrance.fr/beh/2023/1/2023_1_2.html (JEM updated version used)
- Marant Micallef C, Charvat H, Houot MT, Vignat J, Straif K, Paul A, et al. Estimated number of cancers attributable to occupational exposures in France in 2017: an update using a new method for improved estimates. J Expo Sci Environ Epidemiol. 2021.(JEM updated version used)
- Houot M-T, Homère J, Goulard H, Garras L, Delabre L, Pilorget C. Lifetime occupational exposure proportion estimation methods: a sensitivity analysis in the general population. Int Arch Occup Environ Health. 2021.
- Lorentz E, Despreaux T, Quignette A, Chinet T, Descatha A. [Screening of occupational exposure to asbestos and silica by job-exposure matrix among patients with lung cancer and mesothelioma]. Rev Mal Respir. 2019;36(10):1088-95.
- El Zoghbi M, Salameh P, Stucker I, Paris C, Pairon JC, Gislard A, et al. Prevalence of occupational exposure to asbestos and crystalline silica according to phenotypes of lung cancer from the CaProMat study: A case-only study. Am J Ind Med. 2018;61(1):85-99.
- Radoi L, Barul C, Menvielle G, Carton M, Matrat M, Sanchez M, et al. Risk factors for salivary gland cancers in France: Results from a case-control study, the ICARE study. Oral Oncol. 2018;80:56-63.
- El Zoghbi M, Salameh P, Stucker I, Paris C, Pairon JC, Gislard A, et al. Phenotypes of lung cancer and statistical interactions between tobacco smoking and occupational exposure to asbestos and crystalline silica from a large case-only study: The CaProMat study. Lung Cancer. 2017;112:140-55.

- Florentin A. Matrices emplois-expositions et émergence des risques professionnels: application au sein du Réseau National de Vigilance des Pathologies Professionnelles Université de Lorraine; 2017.

- Florentin A, Zmirou-Navier D, members RP, Paris C. Contribution of job-exposure matrices for exposure assessment in occupational safety and health monitoring systems: application from the French national occupational disease surveillance and prevention network. Int Arch Occup Environ Health. 2017;90(6):491-500
- Homere J, Goulard H, Audignon Durand S, Delabre L, Pilorget C. Exposition professionnelle aux poussières alvéolaires de silice cristalline libre des artisans retraités du régime social des indépendants. Programme ESPrI. Estimation de la prévalence et de la durée d'exposition vie entière. Saint-Maurice: Institut de veille sanitaire; 2014.
- Paget-Bailly S. Facteurs de risque professionnels des cancers des voies aéro-digestives supérieures : Synthèse des données épidémiologiques et analyse d'une étude cas-témoins, l'étude Icare Université Paris Sud - Paris XI; 2012.

- Lacourt A, Gramond C, Audignon S, Ducamp S, Fevotte J, Soit Ilg AG, et al. Pleural mesothelioma and occupational coexposure to asbestos, mineral wool, and silica. Am J Respir Crit Care Med. 2013;187(9):977-82.

- Paget-Bailly S, Guida F, Carton M, Menvielle G, Radoi L, Cyr D, et al. Occupation and head and neck cancer risk in men: results from the ICARE study, a French population-based case-control study. Journal of occupational and environmental medicine. 2013;55(9):1065-73.

- Fevotte J, Dananche B, Delabre L, Ducamp S, Garras L, Houot M, et al. Matgene: a program to develop job-exposure matrices in the general population in France. Ann Occup Hyg. 2011;55(8):865-78.
- Groupe de travail Matgéné. Présentation d'une matrice emplois-expositions aux poussières alvéolaires de silice cristalline libre – Quelques applications à un échantillon de population en France. Saint-Maurice (Fra) : Institut de veille sanitaire, décembre 2009, 6 p. Available on : www.exp-pro.fr
